# Characterization of Bacterial Cellulose-Based Wound Dressing in Different Order Impregnation of Chitosan and Collagen

**DOI:** 10.3390/biom10111511

**Published:** 2020-11-03

**Authors:** Khatarina Meldawati Pasaribu, Saharman Gea, Syafruddin Ilyas, Tamrin Tamrin, Izabela Radecka

**Affiliations:** 1 Postgraduate School, Department of Chemistry, Faculty of Mathematics and Natural Sciences, Universitas Sumatera Utara, Jl. Bioteknologi No.1, Medan 20155, Indonesia; khatarinameldawati@yahoo.co.id; 2 Department of Chemistry, Faculty of Mathematics and Natural Sciences, Universitas Sumatera Utara, Jl. Bioteknologi No. 1, Medan 20155, Indonesia; tamrin@usu.ac.id; 3 Cellulosic and Functional Materials Research Centre, Universitas Sumatera Utara, Jl. Bioteknologi No.1, Medan 20155, Indonesia; syafruddin6@usu.ac.id; 4 Department of Biology, Faculty of Mathematics and Natural Sciences, Universitas Sumatera Utara, Jl. Bioteknologi No. 1, Medan 20155, Indonesia; 5 Wolverhampton School of Sciences, Faculty of Science and Engineering, University of Wolverhampton, Wulfruna Street, Wolverhampton WV1 1LY, UK; i.radecka@wlv.ac.uk

**Keywords:** bacterial cellulose, chitosan, collagen, impregnation, wound dressing

## Abstract

Bacterial cellulose (BC), chitosan (Chi), and collagen (Col) are known as biopolymers which have met some properties that are required as wound dressing. This study focused on investigating the fabrication of BC-based wound dressing with chitosan and collagen, since chitosan has red blood cells binding and anti-bacterial properties, while collagen can support cell and tissue growth for skin wounds. The BC-based wound dressing was prepared by impregnating BC fibers in the chitosan and/or collagen solution for 24 h. FTIR was used to confirm the intermolecular interaction of amine and hydroxyl group of chitosan and/or collagen in BC-based wound dressing. Furthermore, the XRD diffractogram of the wound dressing show broader peaks at 14.2°, 16.6°, and 22.4° due to the presence of chitosan and collagen molecules in BC fibers. These results were then supported by SEM images which confirmed that chitosan and collagen were well penetrated into BC fibers. TGA curves revealed that BC/Chi/Col has better thermal properties based on the T_max_ compare to BC/Col/Chi. Feasibility of the mats to be applied as wound dressing was also supported by other tests, i.e., water content, porosity, and hemocompatibility, which indicates that the wound dressing is classified as nonhemolytic materials. However, BC/Col/Chi was considered a more potential wound dressing to be applied compared to BC/Chi/Col since it has larger pores and showed better antibacterial properties (larger zones of inhibition) against *S. aureus* and *E. coli* via disk diffusion tests.

## 1. Introduction

Wounds, whether acute or chronic, can cause tissue damage and, therefore, require special treatment to stimulate the healing process. Different types of wound dressing can be applied to facilitate this process [1,2,3]. Ideally, dressings not only should cover and protect the affected area, but also create optimal environment at the wound site to help healing [4,5].

Cheap, conventional, barrier-type dressings such as gauze or cotton are designed to provide protection to the wounds from external trauma and contamination. Unfortunately, these dressings do not actively enhance the healing process. Recently, several studies carried out in the area of wound management, indicated that a good dressing must play an active role in the healing process [5,6,7,8]. Other research reported that an active wound dressing is not only required to be able to cover the wound in order to avoid contamination and infection but it must also be able to provide sufficient air circulation and removal of wound exudate. Moreover, an active wound dressing is also expected to have other properties, such as an appropriate drug delivery system, and have antimicrobial and non-hemolytic properties [6,9,10]. In addition, the active wound dressing not only needs to effectively accelerate the healing time but also should reduce pain [11,12].

Lately, wound dressings made of natural biopolymers have been extensively studied due to their beneficial properties such as: being non-toxic; easy to be processed; and found in abundance in nature. In addition, the wound dressings which are made of biopolymers show better results for either in vitro or in vivo compatibility [13,14,15]. Many papers have described bacterial cellulose (BC) as an ideal biopolymer that can be used for wound dressing purposes [4,5,16,17]. The chemical structure of BC consists of 1-d-anhydroglucopyranosis chains bounded by β-glycosidic bonds. In addition, the BC structure also involves intra- and inter-molecular hydrogen bonding and van der Waals interactions [13]. This intra- and inter-molecular hydrogen bonding and van der Waals interactions that provide BC with advantages as template which can be impregnated with other materials to improve its properties as required [1,18]. In addition, three-dimensional (3D) dense structure of BC, with neatly woven fibers, provides a high water holding capacity which can give the wound dressings their soft and malleable texture, and which can help to reduce pain in wound dressing application. BC also offers flexibility, high water retention capability, good gas exchange, and provides an excellent physical barrier for microbial pathogens [19].

The success of BC in wound dressing applications is evidenced by the emergence of various BC-based commercial products with such trademarks such as Biofill^®^, Bionext^®^, Dermafill™ Membracell^®^, Prima cel™ and Xcell^®^. It was also reported that BC-based wound dressings show faster epithelialization and tissue regeneration rates in wound-healing treatments compared with conventional gauze or synthetic materials such as Tegaderm^®^, Xeroform™ or Cuprophan^®^ [16].

However, BC itself does not possess any bioactivity performance, i.e., antibacterial and antifungal properties. In addition, BC’s native structure cannot hold cell attachment on its 3D fiber surface due to its high density structure. In order to improve BC performance and to display active wound dressing features, two other biopolymers, chitosan and collagen, were chosen to be impregnated into BC fibers. There are several research papers that report the utilization of both biopolymers in medical applications, such as wound dressings, scaffolds, and/or drug delivery systems [14,20].

Chitosan, (1→4)-linked 2-amino-2-β-deoxy-d-glucopyranose, was considered as a wound dressing material because of its biocompatibility, non-toxicity, biodegradability, and broad antimicrobial spectrum against the growth of bacteria and fungi [21]. Additionally, it displayed binding properties with red blood cells which allow the blood to clot quickly. Furthermore, chitosan was found to play a significant roles in inflammatory cells, and promote granulation [22]. Collagen is known as one of the main proteins in the skin, which is biocompatible and can support cell and tissue growth [23]. Therefore, collagen has become a promoting candidate for wound dressing material, since active wound dressings must be chemically similar to a protein structure that can support the proliferation, and enhance fibroblast cell growth, in human skin [24,25,26].

Previous research is limited in fabricating BC mats with chitosan or collagen. Noh et al., reported the use of BC/collagen for wound dressing or scaffolding on human mesenchymal stem cells [23]. Another study by Jia et al., reported the use of BC/chitosan bio-hydrogel for potential scaffolding in tissue engineering [27]. In addition, the literature also reports the possible use of chitosan/BC mats to prepare a potentially flexible platform for wound dressing or scaffolding [28]. Although BC with either chitosan or collagen were used in the previous studies to manufacture wound dressing mats using solution impregnation method, to our knowledge there are no previous reports where all three biopolymers BC, chitosan, and collagen were used together to make a wound dressing with novel, improved properties. Thus, there is no report that shows the effects of different orders in ex situ chitosan and collagen impregnation on BC.

In this study, a BC impregnated with chitosan and collagen dressing was expected to improve BC properties, since chitosan and collagen are known to have different chemical properties but can complement each other and interact chemically through hydrogen bonding without any significant change in the chemical properties. Therefore, they will express their characteristics for wound dressing individually [27,28,29,30,31]. It becomes important to optimize the impregnation order of the resulting system. The impregnation process was also confirmed and can improve BC antibacterial properties and hemocompatibility properties.

## 2. Materials and Methods

### 2.1. Materials

Coconut water was obtained from local markets in Medan, Indonesia. Bacterial cellulose producer strain of *A. xylinum* was obtained from the Material and Polymer Lab, Postgraduate Chemistry, Universitas Sumatera Utara, Medan, Indonesia.

Pure fish collagen peptide was purchased from Delisari Nusantara, Jakarta, Indonesia. Chitosan (DDA 73.72%, MW 331.131 kDa) was purchased from the Center of Excellence Chitosan and Advanced Material, Universitas Sumatera Utara, Medan, Indonesia. While glucose, urea, glacial acetic acid and NaOH were purchased from Merck (Damstadt, Germany) and used without any further purification.

### 2.2. The Preparation of BC

BC production was carried out using coconut water as the medium, which was supplemented with glucose (10 g/L) and urea (5 g/L) as carbon and nitrogen sources, respectively. Glacial acetic acid was added to adjust the pH of medium to 4.5 [32]. The medium was then sterilized at a temperature of 121 °C for 15 min. The resulted medium was inoculated with 10 mL of *A. xylinum* starter culture, and incubated at room temperature (between 25 and 30 °C) for seven days. During the incubation, cellulose fibers were secreted by *A. xylinum* in the form of gel. After seven days, the obtained gel was harvested and purified by immersing in 2.5% NaOH for 24 h. Finally, pure BC gels were washed with distillated water until neutral pH was achieved [33].

### 2.3. The Preparation of Wound Dressing

**Figure 1:**
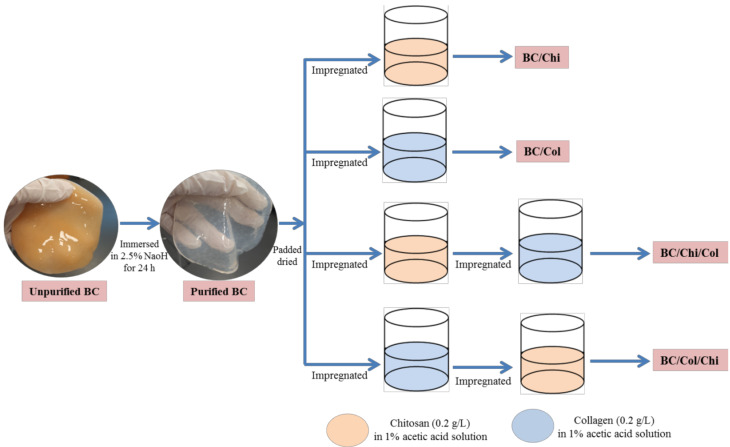
Schematic illustration of the BC-based wound dressing fabrication process.

Pure BC gels with a diameter surface about 80 mm and a thickness about 3 mm were padded dried. Each dried BC gel was placed into 100 mL of chitosan solution (0.02 g/mL powder in 1% acetic acid solution) for 24 h under 100 rpm stirring condition at room temperature to form BC/Chi. The BC/Chi gel was formed then patted dry before impregnating in 100 mL of collagen solution (0.02 g/mL powder in 1% acetic acid solution) for 24 h under stirring condition. The formed wound dressing was lyophilized for 72 h using a freeze dryer. The final prepared sample was labeled as BC/Chi/Col. The other wound dressings, i.e., BC/Chi, BC/Col, and BC/Col/Chi, were prepared as shown in Figure 1. Samples were stored in a humidity chamber to maintain their moisture content until further use.

### 2.4. Determination of Impregnated Chitosan or Collagen Percent Weight

The amount of impregnated collagen or chitosan was calculated by comparing the difference of chitosan or collagen solution volume before and after the impregnation process, as shown in Equation (1):

(1) $$Impregnated\;chitosan\;or\;collagen\;\left(\;\%\;\right)\frac{w}{w}=\frac{\left(V_{1}-V_{2}\right)\;\mathrm{mL}\;\times \;0.02\frac{\mathrm{gr}}{\mathrm{mL}}}{Lyophilized\;BC\;weight}\times 100\%$$

where:

*V*_1_ = Initial chitosan/collagen solution volume
*V*_2_ = Chitosan/collagen solution volume after impregnation

### 2.5. Fourier Transforms Infrared (FTIR)

The functional groups of lyophilized samples were investigated using Fourier transform infrared (FTIR) spectroscopy. The sample was pulverized and mixed with KBr (1 mg/100 mg) and analyzed in a wavenumber range of 500–4000 cm^−1^ with 64 scans. It was recorded using a Shimadzu 8201PC FTIR spectrophotometer (Shimadzu, Tokyo, Japan) at Gadjah Mada University, Indonesia.

### 2.6. X-Ray Diffraction (XRD)

In order to determine the crystallinity of the samples, X-Ray Diffraction (XRD) was carried out using Shimadzu XRD-6100 (Shimadzu, Tokyo, Japan) diffractometer at Medan State University, Indonesia. In this study, the calculation of the crystallinity index was based on the equation as proposed by Osorio-Madrazo et al. [34].

(2) $$CrI\;\left(\%\right)=\frac{S_{c}}{S_{t}\;}\times 100$$

where:

*S_c_* = Area of the crystallinity
*S_t_* = Area of total domain

### 2.7. Thermal Gravimetric Analysis (TGA)

Thermal stability of the sample was measured by thermal gravimetric analysis (TGA) and derivative thermal gravimetric analysis (DTGA) using an EXTAR 7300 Series (Netzsch, Selb, Germany) at the University of Lampung, Indonesia. The sample was heated with heating rate of 10 °C/min at the temperature range from 30–600 °C under an atmospheric nitrogen condition.

### 2.8. Scanning Electron Microscope (SEM)

SEM to determining the morphological surface of samples were coated with gold and carried out using SEM EDX EVO MA Zeiss Bruker (Zeiss, Cambridge, UK) at Mabes Polri, Indonesia, operated at 20 kV for chitosan and collagen samples and at 30 kV for BC and BC-based wound dressings. Pore diameters were calculated from images by ImageJ software (https://imagej.nih.gov/ij/download.html) (NIH, Betesda, MD, USA). The diameters were given as the average ± standard deviation.

### 2.9. Moisture Content (MC)

The BC and BC based wound dressing ability to bind water was determined by Equation (3). The statistical result was determined using one-way ANOVA, and followed by post-hoc test using Dunnett’s method. Normally, *p* < 0.05 is used to show the significant result.

(3) $$MC\;\left(\%\right)=\frac{W_{w}-\;W_{d}}{W_{w}\;}\times 100$$

where:

*W_w_* = recorded wet mass of samples before lyophilized
*W_d_* = recorded dry mass of samples after lyophilized

### 2.10. Antimicrobial Properties

The antimicrobial activity of BC, BC/Chi, BC/Col, BC/Chi/Col, and BC/Col/Chi was tested using disc diffusion in which the wound dressing was punched to form a disc with an 8 mm diameter. Mueller–Hinton agar plates (Merck, Germany), swabbed with a Gram-positive *Staphylococcus aureus* or a Gram-negative *Escherichia coli* bacterial strains using sterile cotton to form an even lawn. All work was performed in a laminar airflow, and cultures were then incubated at 37 °C for 24 h. The formed zone of inhibition (ZOI) was measured and analyzed using two-way ANOVA in GraphPad Prism 8 with a Tukey multi-compare test, *p* < 0.05, normally.

### 2.11. Porosity Assessment

The porosity of lyophilized purified BC and BC-based wound dressing was measured by immersing the sample in ethanol (96%) for 10 min and calculated by Equation (4):

(4) $$Porosity\;\left(\%\right)=\frac{V_{1}-V_{3}}{V_{2}-V_{3}\;}\times 100$$

where:

*V*_1_ = Initial volume of ethanol
*V*_2_ = Volume of ethanol when sample was immersed
*V*_3_ = Volume of ethanol after the sample was taken

### 2.12. Hemocompatibility Testing

Hemocompatibility testing was conducted by shaping BC and BC-based wound dressings into an 8 mm disk using a biopsy punch. Anticoagulant horse blood was washed in 0.9% saline and centrifuged at 3000 rpm for 10 min using MPW-150R (MPW Med. Instruments, Warsaw, Poland) before being re-suspended in saline solution. Samples were then soaked in the suspended horse blood cells for 2 h at 37 °C under agitation conditions. Normal saline was served as negative controls and distilled water was used as positive controls. The percentage of hemolysis was determined at a wavelength of 540 nm using Shimadzu 1800 which is indicates proportional concentration of free hemoglobin in water [35]. Finally, it was calculated using Equation (5) and analyzed using two-way ANOVA in GraphPad Prism with a Tukey multi-compare test, and with significance at *p* < 0.05.

(5) $$\mathrm{Hemolysis}\;\left(\%\right)=\frac{A_{s}-A_{nc}}{A_{pc}-A_{nc}\;}\times 100$$

where:

*A_s_* = Absorbance of samples
*A_nc_* = Absorbance of negative control
*A_pc_* = Absorbance of positive control

## 3. Results

### 3.1. Analysis of FTIR

**Figure 2:**
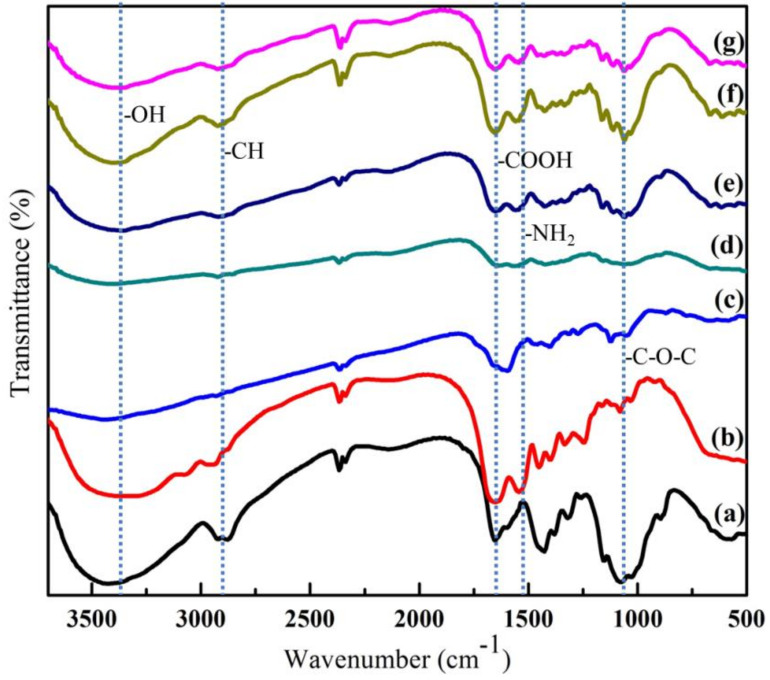
FTIR spectra of (**a**) chitosan, (**b**) collagen, (**c**) bacterial cellulose, (**d**) BC/Chi, (**e**) BC/Col, (**f**) BC/Chi/Col, and (**g**) BC/Col/Chi.

**Figure 3:**
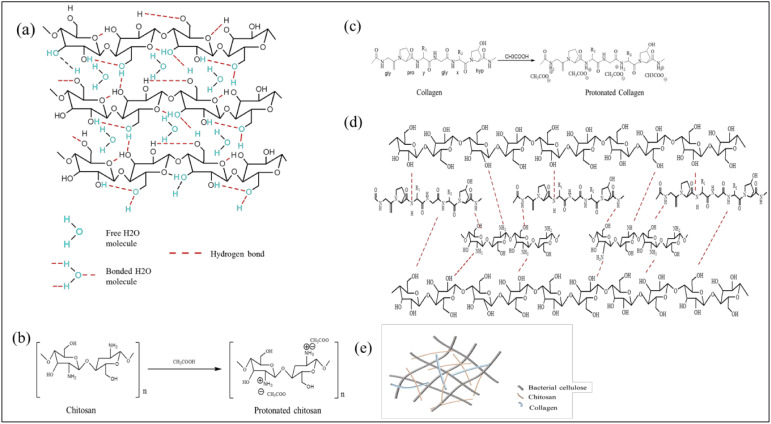
Illustration of (**a**) BC structure; (**b**) protonated chitosan structure; (**c**) protonated collagen structure; (**d**) BC-based wound dressing structure; and (**e**) BC-based wound dressing.

The typical bands of BC functional groups can be clearly seen in Figure 2. An absorption band at 3310 cm^−1^ can be identified as the characteristic band of the O-H stretching vibration in BC. This band was also observed in both BC/Chi and BC/Col mats but it has become broader due to the overlapping of N-H and O-H peaks [28,36,37]. Furthermore, the band at 1650 cm^−1^ was assigned to the amide groups which were specifically found in collagen [31]. This band was also found in BC/Col, BC/Chi/Col, and BC/Col/Chi mats. In addition, band of carboxyl signal at 1560 cm^−1^, assigned as characteristic band of chitosan, was also found in BC/Chi, BC/Chi/Col, and BC/Col/Chi wound dressing mats. However, it can be observed that there was shifting and decreasing intensity of the peak in both the BC/Chi/Col and BC/Col/Chi fabricated mats for wound dressing applications. This was consistent with previous research which demonstrated that the integration of collagen and chitosan may induce hydrogen bonds to form between the two molecules as shown in Figure 3, which shifted and reduced the intensity of amino and carbonyl peaks [38]. A sharp and steep band that was observed at 1080 cm^−1^ is due to the presence of C-O-C stretching vibrations [36]. From the results of FTIR, the intensity of C-O-C in chitosan was higher, compared to the peak intensity of C-O-C contained in collagen. This is also supported by the chemical structure of chitosan and collagen shown in the picture which shows that there are many C-O-C bonds in the chemical structure of chitosan. In BC/Chi/Col, it can be seen that the peak C-O-C intensity is higher than BC/Col/Chi, this indicates that the content of BC-based wound dressing is more influenced by the biopolymer, which was first impregnated into BC. It was also found in the -CH and -COOH peaks, where the peak intensity of -CH was higher at BC/Chi/Col because the first impregnation process was performed on chitosan. In addition, the peak of the -COOH group was found to be higher in BC/Col/Chi because the first impregnation sequence was performed on collagen making the collagen content higher in BC/Col/Chi than in BC/Chi/Col. This was also supported by the data of the percentage weight of chitosan and collagen in the sample, which is displayed in Table 1

### 3.2. Analysis of XRD

**Figure 4:**
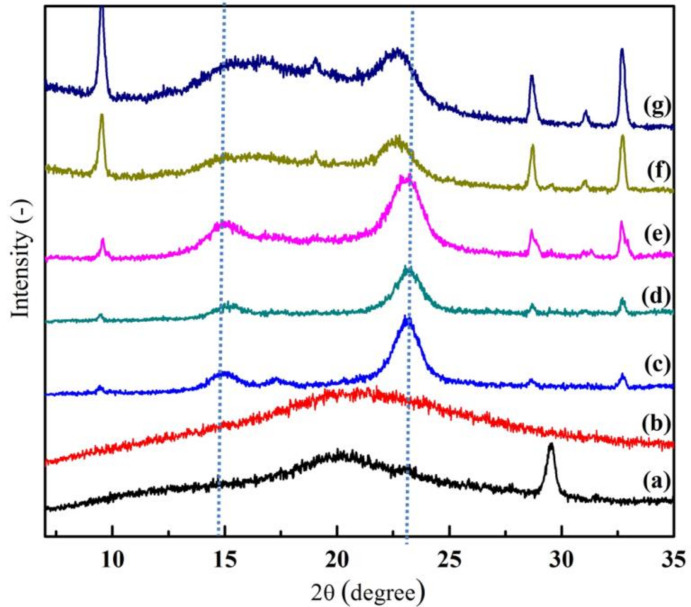
X-Ray diffraction patterns of (**a**) chitosan, (**b**) collagen, (**c**) BC, (**d**) BC/Chi, (**e**) BC/Col, (**f**) BC/Chi/Col, and (**g**) BC/Col/Chi.

From Figure 4, it can be observed that, a crystallographic plane of BC structure was assigned at 14.82° and 23.64°. However, when the other amorphous biopolymers (chitosan and/or collagen) were added to BC, these peaks become broadened and the intensity of peaks decreased. As a result, the crystallinity index (CrI) of prepared wound dressing was reduced [7,36]. Moreover, it can be seen from Table 1 that the CrI number of pure BC at 92.88% was noticeably higher compared to BC/Chi/Col and BC/Col/Chi wound dressing CrI, where it were calculated to be 73.80 and 73.01%. This occurs because the existence of other materials can affect the BC fibers, where the presence of other materials trapped in the BC fibers can enlarge the pore, which affects the decreasing BC crystallinity [27]. Chemically, the presence of chitosan and collagen molecules in the wound dressing also disrupted the hydrogen bonds in BC and, thus, reduced the crystalline area of the mats. Furthermore, the lower number of BC/Col/Chi CrI wound dressing when compared to BC/Chi/Col may occur due to the different impregnation order of chitosan and collagen. The CrI of BC/Col/Chi was lower than BC/Chi/Col because it was impregnated with collagen first, which, from the Table 2, it is observed that collagen is more amorphous than chitosan. CrI should be seriously considered in wound dressing fabrication since high crystallinity could lead to the formation of a microchannel structure, and at the same time the large surface area of the polymer matrix could cause the drug to be released easily [39].

### 3.3. Analysis of TGA/DTGA

**Figure 5:**
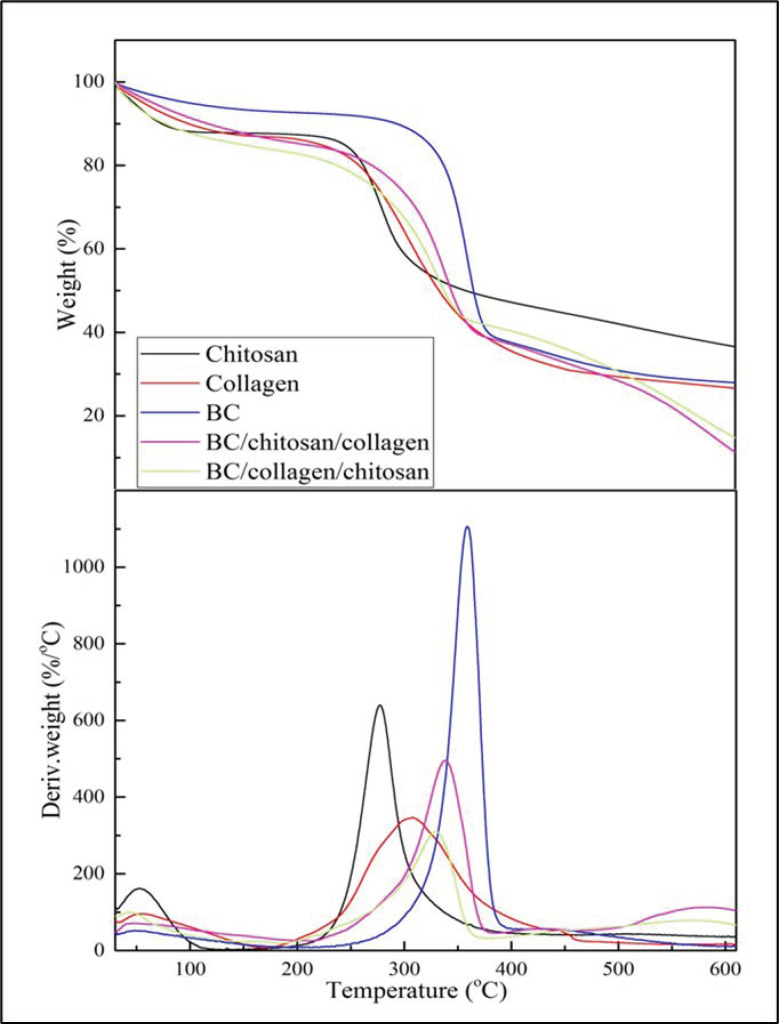
(**a**) TGA and (**b**) DTGA curves.

From the TGA (Figure 5a) and DTGA (Figure 5b) curves, it can be seen that the wound dressing which was produced from BC, chitosan, and collagen biopolymers possessed good thermal properties. However, if the two wound dressings were compared it was found that the BC/Chi/Col wound dressing had better thermal properties compared to BC/Col/Chi. This was correlated with CrI, which has shown BC/Col/Chi has lower CrI than BC/Col/Chi. It was understood that the crystallinity index was one of the structural parameters that could influence the temperature of thermal degradation [40]. The lower CrI of the wound dressing implied the thermal degradation temperature shifted to a lower temperature.

Based on data from Table 3, it can be observed, that the wound dressing thermal properties are more similar to the properties of the last biopolymer that was impregnated into the BC fiber. The BC/Chi/Col wound dressing seems to have T_5_, T_max_, and residual mass of 65.46 °C, 338 °C, and 11.28% respectively as collagen has T_5_, T_max_, and residual mass of 56.76 °C, 305.5 °C, and 26.60 °C, respectively. The same pattern also happened to BC/Col/Chi which has T_5_ of 46.92 °C, T_max_ of 329.0 °C, and residual mass of 14.72%, and chitosan has a value of T_5_ of 47.61 °C, T_max_ of 277.2 °C, and residual mass of 36.56%. T_5_ refers to the temperature while sample experiencing 5% of mass loss and T_max_ refers to the temperature while the sample experiences maximum mass loss.

### 3.4. Analysis of SEM

**Figure 6:**
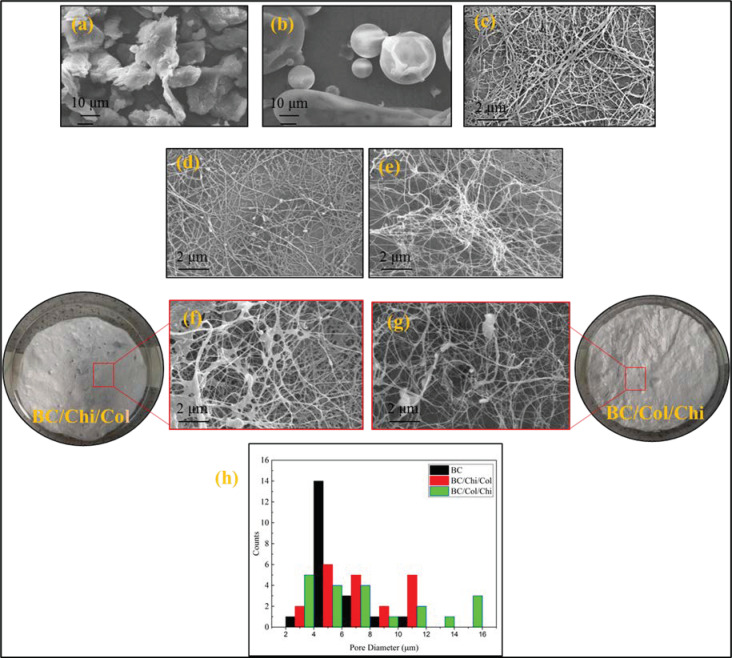
SEM photographs of (**a**) pure chitosan powder, (**b**) pure collagen powder, (**c**) lyophilized pure BC fiber, (**d**) lyophilized BC/Chi mats, (**e**) lyophilized BC/Col mats, (**f**) lyophilized BC/Chi/Col mats, (**g**) lyophilized BC/Col/Chi mats, and (**h**) pore diameter distribution.

From the observation of wound dressing morphology in Figure 6, it can be seen that chitosan and collagen molecules are trapped in BC fibers. Chitosan and collagen molecules changed the porous structure of BC in the wound dressing by enlarging the pore size, although maintaining the same fiber structure. Based on observations of 20 pore areas in the SEM images, it is known that the impregnation process in BC/Col/Chi wound dressing produces a larger pore than BC/Chi/Col. This confirms XRD data which showed wider peak after chitosan and collagen were impregnated and lower CrI values in BC/Col/Chi compare to BC/Chi/Col wound dressing which indicate a larger fiber pore structure. The larger pore of the wound dressing was expected in this study, since it can support its use in the future as a wound dressing because it can accommodate air exchange.

### 3.5. Moisture Content and Porosity

**Figure 7:**
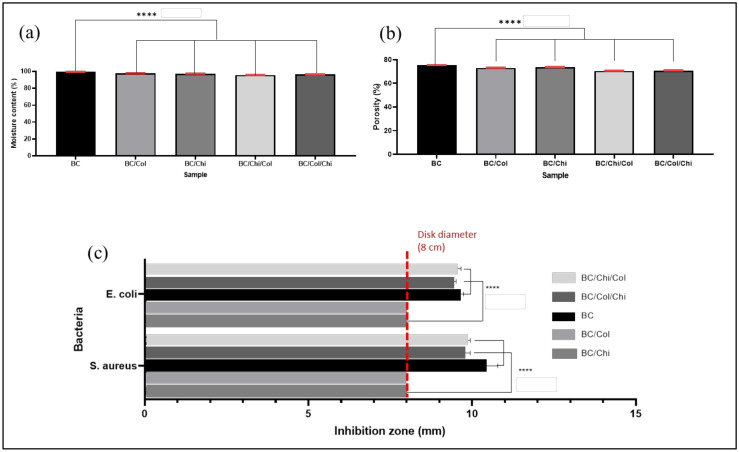
(**a**) Moisture content, (**b**) porosity, and (**c**) antibacterial activity of BC, BC/Col, BC/Chi, BC/Chi/Col, and BC/Col/Chi (*n* = 4) (**** *p* < 0.0001).

BC is known to have the ability to bind water very well; however, the loading of chitosan and collagen into BC reduces the percentage of the moisture content of the wound dressing slightly. From the calculation which was displayed in Figure 7a, the percentage moisture content of each sample is 99.6 ± 0.04, 97.8 ± 0.02, 97.5 ± 0.07, 95.9 ± 0.06, and 96.7 ± 0.05 for BC, BC/Col, BC/Chi, BC/Chi/Col, and BC/Col/Chi, respectively. It was known that the different order of impregnation in preparing this wound dressing does not significantly affect the moisture content of the wound dressing.

Based on the calculation of the porosity percentage as displayed in Figure 7b, the results showed that the presence of chitosan and collagen reduces the porosity of wound dressing but not significantly different among the wound dressing. The percentage of sample porosity is 75.5 ± 0.06, 73.2 ± 0.09, 73.8 ± 0.07, 70.7 ± 0.12, and 70.9 ± 0.10% for BC, BC/Col, BC/Chi, BC/Chi/Col, and BC/Col/Chi, respectively. This also indicated that BC, chitosan, and collagen filled the BC pore which caused in decrease in the percentage of porosity.

### 3.6. Antibacterial Activity

Based on antibacterial tests, it is known that BC and collagen do not have antibacterial properties, as seen in Figure 7c. This is consistent with a number of studies [17,41], which also indicated the same result. Chitosan, however, has antibacterial properties as it can prevent cell division in the Gram-positive peptidoglycan bacterial layer of the cell wall by forming non-covalent teichoic acid bonds, while in Gram-negative bacteria, chitosan can disrupt the absorption of nutrients due to its pH above the pKa and form an electrostatic interaction with anionic lipopolysacaride groups at the surface [42]. From the clear zone measurements for *S. aureus*, the results obtained were 2.15 ± 0.13 mm, 1.7 ± 0.10 mm, and 1.95 ± 0.10 mm for BC/Chi, BC/Chi/Col, and BC/Col/Chi, respectively, while the measurement of the clear zone against *E. coli* obtained data were 1.7 ± 0.14 mm, 1.4 ± 0.11 mm and 1.7 ± 0.15 mm for BC/Chi, BC/Chi/Col, and BC/Col/Chi, respectively. Presented results suggest that fabricated BC/Chi and BC/Col/Chi mats show better antibacterial properties (bigger zones of inhibition) than BC/Chi/Col mats due to chitosan being impregnated on BC after collagen and, therefore, present on the surface of the prepared wound dressing mats.

### 3.7. Hemocompatibility

Hemocompatibility testing was carried out to investigate the biocompatibility properties of the wound dressing with blood. This test observed the percentage release of hemoglobin into plasma due to the damage of erythrocytes. The hemocompatibility percentage (%) of all obtained samples include BC/Chi/Col and BC/Col/Chi was found to be less than 2% (Table 4), thus, fabricated dressings can be classified as nonhemolytic materials and can be applied as wound dressings with respect to the ASTM F756-00 standard. The variation in percent hemompatibility observed is related to the order of impregnation in the BC and refers more to the nature of the biopolymer impregnated to the outside surface of the dressing.

## 4. Conclusions

BC/Chi/Col and BC/Col/Chi wound dressings were successfully prepared by an ex situ method using the solution impregnation technique. FTIR results show characteristic bands for functional groups of all three biopolymers: BC, chitosan, and collagen were also found in BC/Chi/Col and BC/Col/Chi fabricated by impregnation of novel dressings. Furthermore, there was a band that showed interaction of amine and hydroxyl groups which confirmed the presence of chitosan and/or collagen in BC-based wound dressing. The broader 2 theta peak in the BC/Chi/Col dressing than in BC/Col/Chi was in line with CrI data, which showed that BC/Chi/Col has a higher CrI than the BC/Col/Chi fabricated dressing. This is further reinforced by the results of the SEM observation, which shows that the BC/Col/Chi mat had larger pores than BC/Chi/Col. In addition, a thermal test using TGA showed that the BC/Chi/Col dressing had better thermal properties than BC/Col/Chi. Performed tests on moisture content, porosity, antibacterial properties, and hemocompatibility resulted in similar results for both mats (BC/Chi/Col and BC/Col/Chi). Therefore, it can be concluded that both BC/Chi/Col and BC/Col/Chi fabricated dressing may have potential application as wound dressings. It can also be concluded that the BC/Col/Chi mat may have greater potential as a wound dressing as it has larger pores and shows better antibacterial activity.

## Figures and Tables

**Table 1:** The weight percentage of impregnated sample.

Sample	BC (Chitosan % Weight)	BC (Collagen % Weight)
BC/Chi	32 ± 1.5	0
BC/Col	0	33 ± 2.3
BC/Chi/Col	32 ± 1.5	10 ± 1.2
BC/Col/Chi	15 ± 1.2	33 ± 2.3

**Table 2:** Crystanillity index (CrI) data of chitosan, collagen, BC, BC/Chi, BC/Col, BC/Chi/Col, and BC/Col/Chi.

Sample	CrI (%)
Chitosan	68.6
Collagen	59.9
BC	92.8
BC/Chi	89.0
BC/Col	89.3
BC/Chi/Col	73.8
BC/Col/Chi	73.0

**Table 3:** T_5_, T_max_, and residual mass of chitosan, collagen, BC, BC/Chi/Col, and BC/Col/Chi.

Samples	T_5_ (°C)	T_Max_ (°C)	Residual Mass (%)
Chitosan	47.6	277.2	36.5
Collagen	56.7	305.5	26.6
BC	98.8	358.4	27.9
BC/Chi/Col	65.4	338.5	11.2
BC/Col/Chi	45.9	329.0	14.7

**Table 4:** Hemocompatibility percentage of BC, BC/Chi, BC/Col, BC/Chi/Col, BC/Col/Chi (*n* = 4).

Sample	Hemocompatibility (%)
BC	1.50 ± 0.18
BC/Chi	1.63 ± 0.05
BC/Col	1.58 ± 0.10
BC/Chi/Col	1.60 ± 0.06
BC/Col/Chi	1.65 ± 0.08
